# Features extraction using encoded local binary pattern for detection and grading diabetic retinopathy

**DOI:** 10.1007/s13755-022-00181-z

**Published:** 2022-06-29

**Authors:** Mohamed A. Berbar

**Affiliations:** Department of Computer Engineering and Sciences, Faculty of Electronic Engineering, Menofia University, Menouf, 32952 Egypt

**Keywords:** Diabetic retinopathy, Histogram matching, Local binary patterns, Fundus image, SVM, CNN

## Abstract

**Introduction:**

Reliable computer diagnosis of diabetic retinopathy (DR) is needed to rescue many with diabetes who may be under threat of blindness. This research aims to detect the presence of diabetic retinopathy in fundus images and grade the disease severity without lesion segmentation.

**Methods:**

To ensure that the fundus images are in a standard state of brightness, a series of preprocessing steps have been applied to the green channel image using histogram matching and a median filter. Then, contrast-limited adaptive histogram equalisation is performed, followed by the unsharp filter. The preprocessed image is divided into small blocks, and then each block is processed to extract uniform local binary patterns (LBPs) features. The extracted features are encoded, and the feature size is reduced to 3.5 percent of its original size. Classifiers like Support Vector Machine (SVM) and a proposed CNN model were used to classify retinal fundus images. The classification is abnormal or normal and to grade the severity of DR.

**Results:**

Our feature extraction method was tested on a binary classifier and resulted in an accuracy of 98.37% and 98.84% on the Messidor2 and EyePACS databases, respectively. The proposed system could grade DR severity into three grades (0: no DR, 1: mild DR, and 5: moderate, severe NPDR, and PDR). It obtains an F1-score of 0.9617 and an accuracy of 95.37% on the EyePACS database, and an F1-score of 0.9860 and an accuracy of 97.57% on the Messidor2 database. The resultant values are dependent on the selection of (neighbours, radius) pairs during the extraction of LBP features.

**Conclusions:**

This study’s results proved that the preprocessing steps are significant and had a great effect on highlighting image features. The novel method of stacking and encoding the LBP values in the feature vector greatly affects results when using SVM or CNN for classification. The proposed system outperforms the state of the artwork. The proposed CNN model performs better than SVM.

## Introduction

Diabetic Retinopathy (DR) is a severe microvascular complication of diabetes disease. DR is diagnosed by detection of lesions, haemorrhages, microaneurysms (MAs), and exudates [1, 2] or diagnosed directly without lesions segmentation by detecting abnormalities [3]. Haemorrhages (HEM) are mostly caused by the leakage of weak vessels and are characterized as big red spots. Micro aneurysms (MAs) are round, small, and have a dark red colour. Exudates are the third symptoms of DR that are yellowish, irregular in shape, and shiny. Exudates are due to the leaking of lipoproteins and proteins out of the retinal vessels. MAs are the earliest features that could be detected in DR. MAs can cause blockage of blood vessels in the retina and may herniate, causing haemorrhages. MAs are features that can always be detected in the early stages, while haemorrhages can be found in more advanced stages. Many computerised DR detection methods are suggested for the detection and removal of optical disc (OD) (Rathod et al. [4]; Sekar and Nagarajan [5], Lu and Lim [6], Acharya et al. [7], Trucco et al. [8]) because its colour is the same as the other features of retinal images like exudates. The rapid increase of diabetic patients requires the development of diagnostic systems. These systems are necessary to help with the manual diagnosis of related issues. Automated detection of DR can be achieved using image processing and machine learning techniques that will computerise the diagnosis process and decision-making. The computerised diagnosis system could be used to sort the DR patient from diabetic patients, and then the doctors could follow him up. Unfortunately, fundus images are suffering from noise. The images in the same database are not of the same brightness or contrast. In this paper, a new method for balancing the contrast and brightness of the funds’ image is proposed. Traditional methods with the segmentation stage are excluded because they introduce the possibility of segmentation stage error propagation to the rest of the DR system. Thus, detecting diabetic retinopathy in retinal images without the need for segmentation of DR components is our research goal, which is a challenging task that needs to be resolved. By looking for a good method for features extraction without the segmentation stage, we found that local binary pattern (LBP) has been applied in numerous pattern recognition systems and shows promising results for medical imaging, such as in breast cancer [9], DR detection [3, 10] and glaucoma [11]. Another extension of the LBP is the uniform LBP, which denotes the number of spatial transitions in a particular pattern. Uniform LBP has a power discrimination ability compared to the original LBP patterns due to the different statistical properties it has and the fact that the non-uniform patterns have a small proportion in comparison to uniform patterns that represent most of the fundamental texture properties. Uniform LBP and statistical features, and transform methods have been implemented and tested. Unfortunately, results from statistical features, wavelet, and discrete cosine transform methods were not promising. This paper proposes a new novel method based on uniform LBP for features extraction to provide a fast and robust computer diagnosis system for DR detection and grading DR severity. The proposed method for feature extraction is named “Uniform Local Binary Pattern Encoded Zeroes (ULBPEZ)”. The proposed method reduced the feature size to 3.5% of its original size. It jumps forward with a classification score of 10% or more. For classification, SVM and the proposed CNN model are used. The SVM classifier and the CNN are trained with 70% of the extracted and encoded features and evaluated using the remaining 30%. This paper also presents a creative way to represent the extracted ULBPEZ features as an image. That image can be classified later by the proposed CNN.

## Related research works

Traditional studies have concentrated on detecting exudates in the retina as in [1] and [12]. Since the exudates appear as white or yellow spots in retinal images, Kumar et al. [1] emphasised the brighter regions by performing gamma correction on both the red and green channels to extend both histograms. The exudates candidate region is then detected using histogram analysis. The method validated only 158 retinal images. The sensitivity for detecting abnormal cases was 88.45%. Rajput and Patil [12] presented a supervised method for identifying and classifying the exudates. The candidate regions of exudates were detected using the k-means clustering technique, which provides a good result when the retinal OD is fully visible and fails if only a portion of the OD is visible. Some researchers confirm the need for pre-processing to improve the image quality [13, 14]. Pazmino et al. [13] presented a method for processing fundus images to improve the visibility of the vascular network. Luangruangrong [14] used contrast limited adaptive histogram equalization (CLAHE) for image enhancement, then optic disk and blood vessel detection followed by classifying exudates using hierarchical fuzzy-c-mean clustering. Wang et al. [15] detect DR stages in their database and the Messidor-1 database by using a modified R-FCN method based on R-FCN method (Dai et al. [16]). Image augmentations were applied. The obtained sensitivity for detecting DR grades was 92.59% in the Messidor-1 database. Their study did not detect exudates and only detected HEM and MAs.

Some researchers like Ramachandran et al. [17], Szegedy et al. [18], Alex et al. [19], Johari et al. [20], and Lam et al. [21] used pre-trained CNN models like GoogleNet and AlexNet. Others who used their proposed CNN architecture included David et al. [22], Gargeya and Leng [23], Li et al. [24, 25], and Shaban et al. [26].

The pre-trained models fixed the input image to a small size of around 224 × 224 while DR images are big-sized images. These CNN models use filter sizes suitable for objects in the ImageNet database and hyperparameters that may be not compatible with the small details in fundus image texture. Resizing DR images to that small size and ignoring the special nature of the medical image may lead to losing important features of small local details. Li et al. [25] used an algorithm based on CNN to extract fundus image features. They replaced the max-pooling layers with fractional max-pooling. Two CNNs with a different number of layers are trained to extract features. The metadata of the image is combined with the extracted features from CNNs. A support vector machine (SVM) classifier is used for classifying the fundus images of the Kaggle dataset into DR grades. Their accuracy was 86.17%. Shaban et al [26] introduced their CNN to distinguish grades of DR into three levels of severity (Level 0. no DR, level 1: (i.e., a combination of mild (grade 1) and moderate (grade 2) Non-Proliferative DR (NPDR)) and level 2: (i.e., a group of severe NPDR (grade 3), and Proliferative DR (PDR) (grade 4)) with an accuracy of 88–89%, a sensitivity of 87–89%). David et al. [22] used multiple CNNs for the automatic detection of DR lesions on the Messidor-2 dataset. The system outputs three classes: Class 1 (no or mild DR present), class 2: referral DR is present, and class 3: vision-threatening DR is present. Their paper obtained results with 96.8% sensitivity. Gargeya and Leng [23] used a deep CNN model based on deep residual learning. The dataset Messidor-2 was used for evaluation. The model achieved 93% sensitivity as a binary classifier. Katada et al. [27] implemented an AI model for grading DR images. They trained a CNN and an SVM using a data set of American clinical fundus images. The AI model scored a sensitivity of 81.5% for the American validation data set and a sensitivity of 90.8% for the Japanese data set. Manojkumar et al. [10] presented a DR detection system using LBP. They separated the fundus colour image into RGB channels, and then LBP is applied to each channel for the extraction of LBP features. The statistical features are calculated as mean, standard deviation, entropy, kurtosis, and skewness for each channel of the LBP image. A random forest algorithm is used for classification. Colomer et al. [3] used LBPs and granulometric patterns to extract the texture and morphological features of fundus images. Combinations of these features feed the classifier. With SVM their accuracy range is (82.05%:85.33%) and with Gaussian processes for classification, they got 87.62% of accuracy and 83.48% of sensitivity.

## Databases under processing

### Messidor-2

The proposed system has been proven on fundus images obtained from the original Messidor-2 *Decencière* (2014) [28]. Messidor-1 is excluded after a while of research because it has some repeated samples and some mistaken classifications. Overall, Messidor-2 is an update of Messidor-1. The images within the database have significant variability in colour, illumination, resolution, and quality. It contains 874 examinations (1748 images). Three images were excluded because we didn’t have their classification. The study’s experiments utilised 1745 fundus images, 1012 of which have no DR lesions and 733 of which have DR lesions graded into five grades according to the severity (0: no DR, 1: mild DR, 2: moderate, 3: severe DR, Non-Proliferative DR (NPDR), 4: Proliferative DR (PDR)). The number of images that have lesions is considered small compared to the number of normal images, and they are distributed over the four abnormal grades (1:4). The Messidor-2 database was doubled at the beginning of the research work by adding the vertical mirroring version of the database before processing it to be 3490 images.

### Kaggle EyePACS database

The EyePACS database [29] is also used for the evaluation of the proposed system. It is a large dataset of fundus images taken under a variety of imaging conditions. Each image is graded for the severity of diabetic retinopathy on a scale of 0 to 4 as in the Messidor-2 database. The images have noise and contain artifacts. They may be out of focus, underexposed, or overexposed. The images were collected from multiple clinics using a variety of cameras over an extended period. About 3301 images were selected to be used for the evaluation of our proposed system. The Messidor-2 is relatively much better in quality than the EyePACS database. This may be because it has been created by a fixed camera. The developed code for pre-processing, feature extraction, and classification were written using Matlab-19.

## Methodology

### Pre-processing

**Fig. 1:**
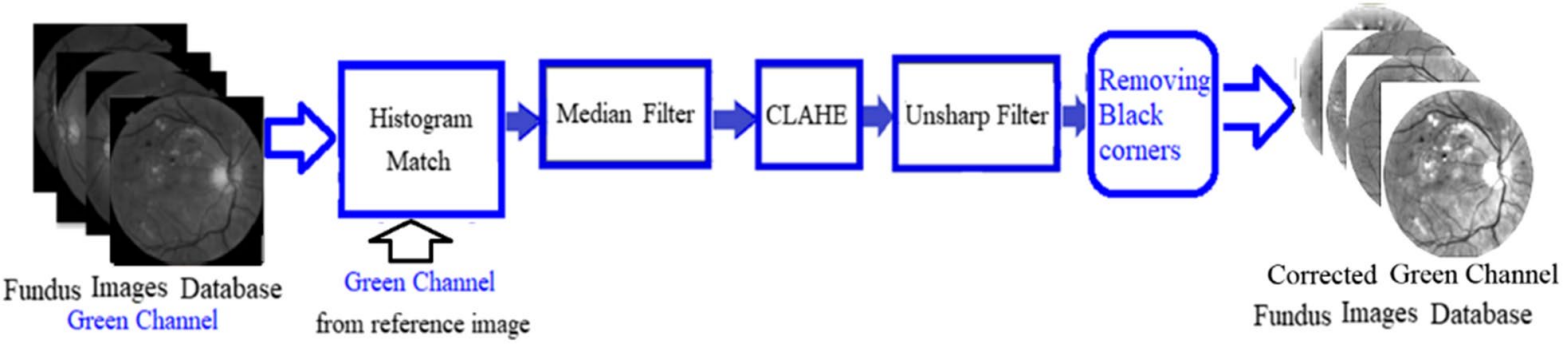
Pre-processing

The pre-processing stage has been carried out and applied before extracting features to standardise the brightness and contrast of the image. Among the RGB channels, the blue channel has the lowest contrast and even suffers from undersaturation and noise, while the red channel suffers from oversaturation [30]. On the other hand, the green channel has the best contrast between the retinal components and the background, providing more structural information [1, 30]. Thus, the study’s proposed method utilises the green channel. Standardization of image quality is achieved when using histogram matching with the selected reference image. A reference image should have balanced brightness and colour, and thus, careful selection is necessary. The output is an image with a brightness that is comparable to that of the reference image. The median filter and contrast limited adaptive histogram equalisation (CLAHE) method are subsequently utilised on the green image, which will be followed by the unsharp filter as shown in Fig. 1.

**Fig. 2:**
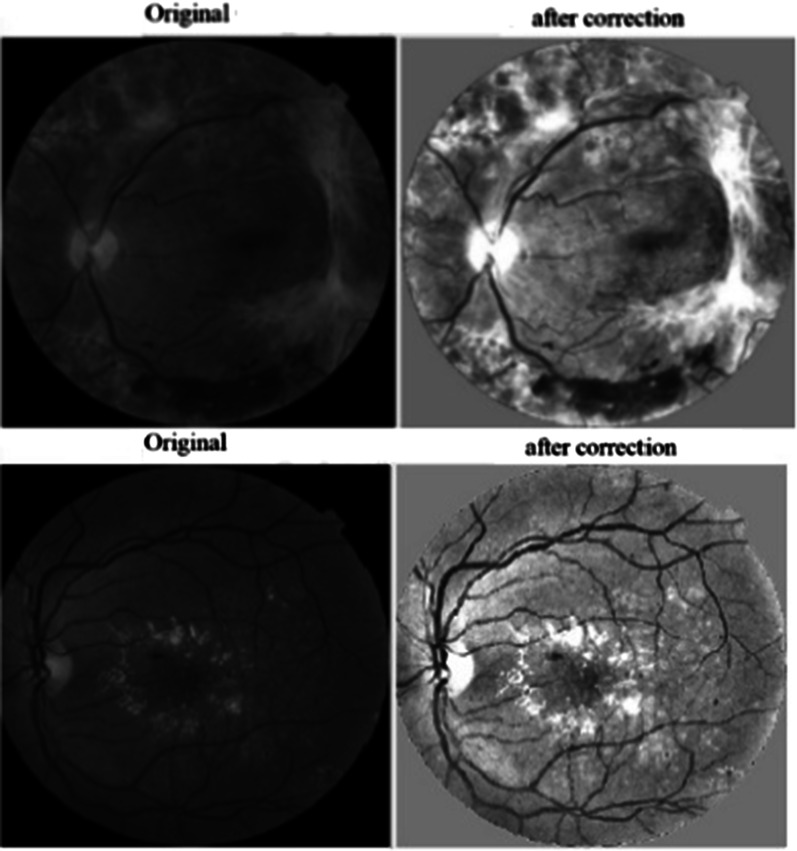
Pre-processing results: original green channel and green channel after correction

CLAHE is used for improving the image contrast and was originally given by Zuiderveld [31]. Figure 2 shows the results of pre-processing. When comparing the original image with the enhanced one, a significant improvement is observed in the information content of the enhanced image.

The black corners are detected, and then the image is divided into blocks of pixels. To avoid redundant data and noisy features not related to the retina itself, any block or part of it belonging to the black background at the corners of the image will be avoided in the features extraction process.

### Features extraction using ULBPEZ method

**Fig. 3:**
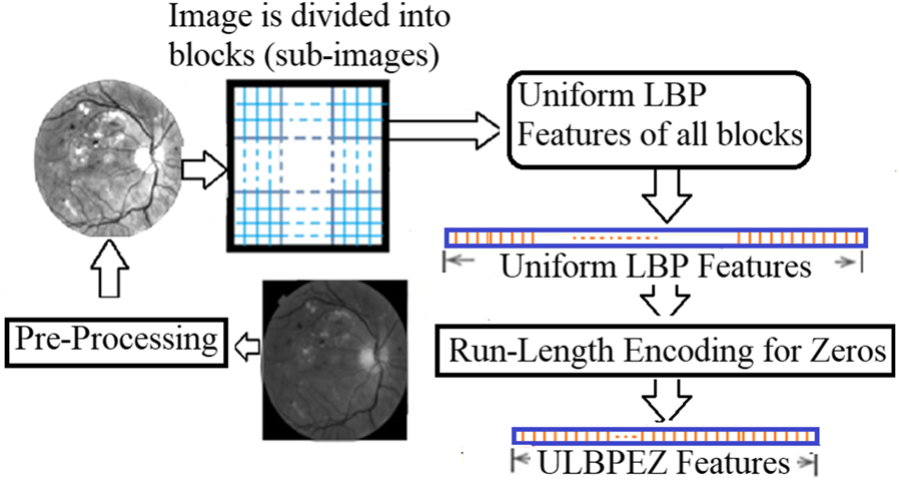
The proposed technique of extracting and forming the encoded uniform LBP (ULBPEZ) features

The fundus image has been resized to a fixed size of 512 × 512 pixels. The images are divided into blocks (sub-images), and each block is processed as an input image for the uniform LBP feature extraction stage. The LBP coefficients are computed based on the circular neighbourhood (P) with radius (R). The uniform LBP features extracted from each block, are concatenated with the previously calculated LBP values from the previous block (see Fig. 3).

**Fig. 4:**
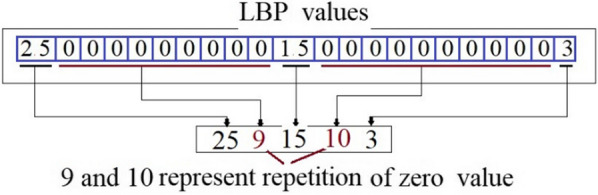
Example to explain proposed technique (ULBPEZ) for encoding zeros values in features vector

After the extraction of the uniform LBP features from all image blocks, the extracted LBP features from all blocks have been stacked, and then the zero coefficients are replaced by encoding them using the theory of the popular RLE algorithm (see Fig. 4). This process reduces the original size of the feature vector to 3.5% of its length before encoding zeros.

The choice of the best block size to be processed for LBP extraction is an important issue. It depends on the smallest lesion in the fundus image and, subsequently, on the image size. Our manual examination of the fundus image reports that the smallest lesion (microaneurysm) is about 5 × 5 pixels at an image size of 512 × 512. A microaneurysm has a small size which constitutes less than 1% of the fundus image reported by Sarhan et al. [32]. The block size should be small to match the smallest lesion, so, our experiments started testing using R = 2 and a block size 5 × 5 pixels. It is not recommended to increase the block size more than necessary to avoid losing local details. Experimental results also prove this recommendation. The block size should be just surrounding the pixel’s neighbours to the processing pixel. The size of the block should be greater than (2 × radius (*R*)) of the circular neighbourhood (see Eq. 1). Then the image divisions into blocks are calculated according to Eq. 2.

1 $$Block\,size = 2 \times R + 1$$

2 $$Divisions = 5 \times floor\left( {image\,size/\left( {5 \times Block\,size} \right)} \right)$$

Originally, the number of neighbours *P* had a great effect on the number of returned LBP coefficients for each block (see Eq. 3).

3 $${\text{Number}}\,{\text{of}}\,{\text{uniform}}\,{\text{LBP}}\,{\text{coefficients}}\,{\text{for}}\,{\text{each}}\,{\text{block = }}P \times \left( {P - 1} \right) + 3$$

The proposed LBP encoded zeros method changed this situation, and P no longer has an effect on the size of the features vector because of encoding zero values. The main parameter affecting feature vector size is radius R in inverse proportion. Increasing R will increase the block size, which will decrease image divisions and the number of blocks per image to be processed and, consequently, the number of extracted features. This research avoids redundant data, any block or part that belongs to the black background at the corners of the image will be masked out in the features extraction process. This is also considered a reduction of feature vector size. The extracted features vector is reduced in size again after encoding zero values. For example: Dividing a 512 × 512 image into 70 × 70 blocks with (P, R) = (8, 3) and block size 7 × 7, will produce a feature vector size of 233581 coefficients after ignoring the blocks belonging to or touching the background at the corners. The feature vector size became 8200 values after encoding zeros at most. Experimentally, after getting rid of zeros in the features vector, the size of the features is found to be at its maximum, as shown in the following Table 1.

**Table 1:** Effect of radius R on features size

R	Block size	Divisions	Features size
2	5 × 5	100 × 100	16,150
3	7 × 7	70 × 70	8200
4	9 × 9	55 × 55	4950
5	11 × 11	45 × 45	3300

### The proposed CNN model for ULBPEZ

The pre-trained GoogleNet, ResNet-50, VGG-19, and AlexNet models fixed the input image to 224 × 224 × 3. We used the pre-trained models with resizing fundus colour images to 224 × 224 × 3 without feature extraction steps. Unfortunately, the resultant accuracy range was 52:65%. Applying a histogram match with a good quality reference image on the red and blue channels of all images of the Messidor-2 dataset improves the resultant accuracy range to (72:75) which is not acceptable for us, and we decided to construct our own CNN model.

**Fig. 5:**
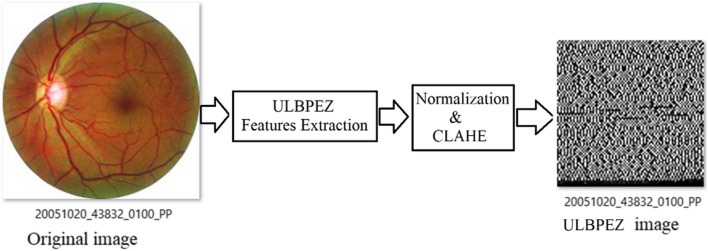
Original image and ULBPEZ image

The extracted vector of ULBPEZ values has been reformed in a square matrix, then normalised and represented as a square image, and each ULBPEZ value is represented as a pixel. The image size was 91 × 91 in the case of R = 3. CLAHE is used to enhance the ULBPEZ image contrast. This process resulted in a new form of database of ULBPEZ images representing the original fundus images (see Fig. 5). The ULBPEZ images have been used with our designed CNN architecture. The analysis of the proposed CNN model is shown in Table 2. All the filters used in the convolution layers are of size [1 5] as 1-D filters with stride 1 and padding 1. The number of filters in Conv1, Conv2, and Conv3 were 8, 16, and 8 respectively. Max Polling layers are used with stride 2 and padding 0. The CNN was trained with 70% of the samples in the newly formed database and tested the remaining 30%.

**Table 2:** The analysis of the proposed CNN for classifying the ULBPEZ image

	Name	Type	Activations	Learnable
1	Image input	Image	91 × 91 × 1	–
2	Conv 1: 8 1 × 5 × 1 convolution with stride [1 1] padding [1 1 1 1]	Convolution	93 × 89 × 8	Weights 1 × 5 × 1 × 8 Bias 1 × 1 × 8
3	Batchnorm1 batch normalization with 8 channels	Batch Normalization	93 × 89 × 8	Offset 1 × 1 × 8 Scale 1 × 1 × 8
4	Relu_1	ReLU	93 × 89 × 8	–
5	Max pool 1: [1 × 2] max pooling with stride [2 2] and padding [0000]	Max Pooling	47 × 44 × 8	–
6	conv2: 16 1 × 5 × 8 convolution with stride [1 1] padding [1 1 1 1]	Convolution	49 × 42 × 16	Weights 1 × 5 × 8 × 16 Bias 1 × 1 × 16
7	Batchnorm2: batch normalization with 16 channels	Batch Normalization	49 × 42 × 16	Offset 1 × 1 × 16 Scale 1 × 1 × 16
8	Relu_2	ReLU	49 × 42 × 16	–
9	Max pool 2: [1 × 2] max pooling with stride [2 2] and padding [0000]	Max Pooling	25 × 21 × 16	–
10	Conv 3: 8 1 × 5 × 1 convolution with stride [1 1] padding [1 1 1 1]	Convolution	27 × 19 × 8	Weights 1 × 5 × 16 × 8 Bias 1 × 1 × 8
11	Batchnorm_3: batch normalization with 8 channels	Batch Normalization	27 × 19 × 8	Offset 1 × 1 × 8 Scale 1 × 1 × 8
12	Relu_3	ReLU	27 × 19 × 8	–
13	Maxpool_3: 1 × 2 max pooling with stride [2 2] and padding [0000]		14 × 9 × 8	
14	fc_1	Fully connected layer	1 × 1 × 100	Weights 100 × 1008, Bias 100 × 1
15	fc_2	Fully connected layer	1 × 1 × 5	Weights 5 × 100, Bias 5 × 1
16	SoftMax	Softmax	1 × 1 × 5	–
17	Class output	Classification Output	–	–

### Evaluation metrics

**Fig. 6:**
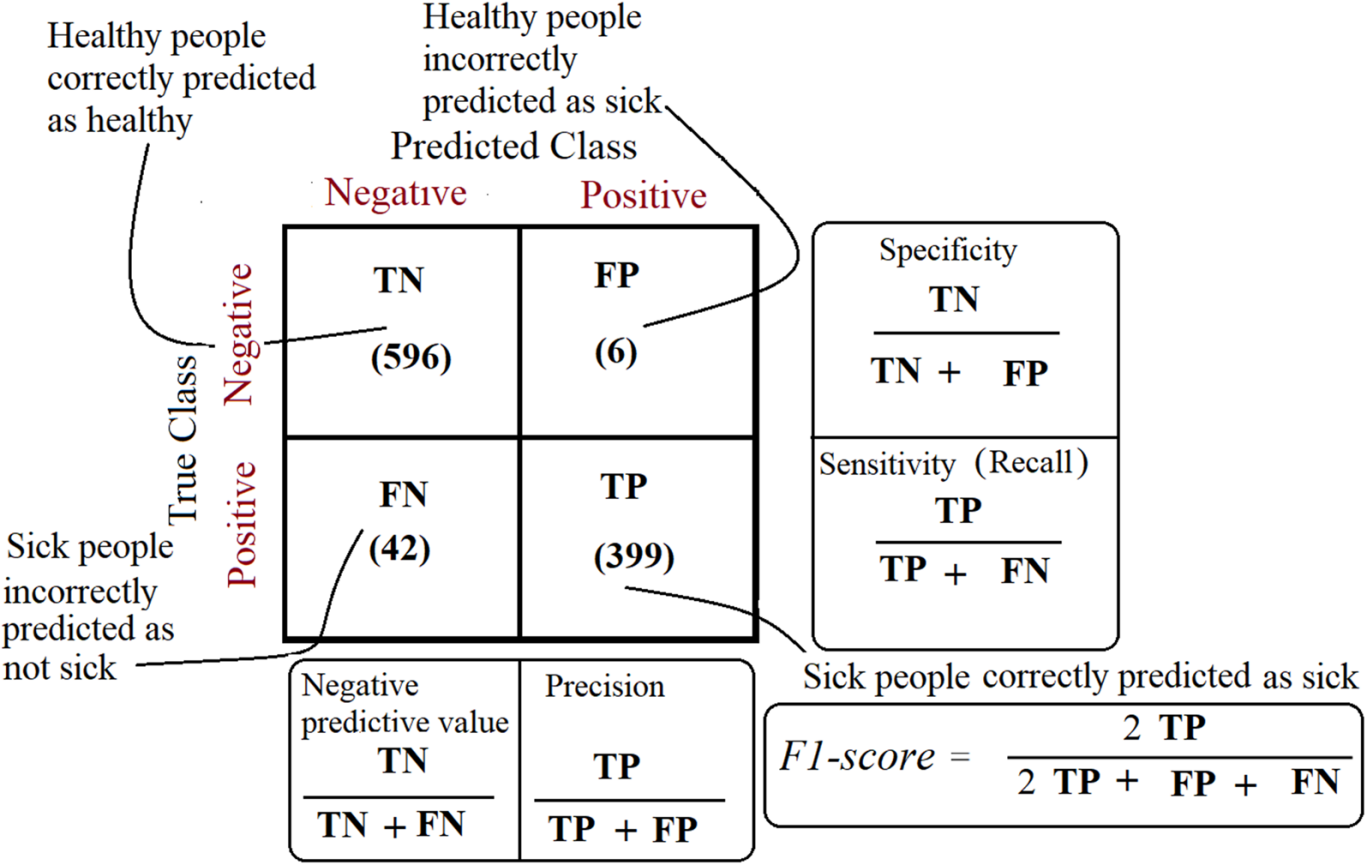
Confusion matrix

The confusion matrix is used for performance evaluation (see Fig. 6). The confusion matrix gives a clear measure to the system, and it does not mislead by only the total accuracy measure, especially when the number of classes is unbalanced as in our datasets under processing.

## Results of binary classifier

In the beginning, we applied uniform LBP on the standard database Messidor-2 to extract features without using ULBPEZ in the extracted features stream. The resultant accuracy (Acc) was 85.1%, and the recall (sensitivity) was bad at about 71.7% with an F1 score of 0.8051. The proposed method ULBPEZ has been applied as local descriptors of each block (sub-image). All series of zeros in the features vector are encoded and the size of the features vector is reduced to 3.5% of its original size. Different values of R (2, 3, 4, 5) are tested with their corresponding block size (5 × 5, 7 × 7, 9 × 9, 11 × 11) with different neighbours P (8, 12, 16, 20, 24) to find the pair (P, R) giving the best recall and F1-score. The features extracted using ULBPEZ are tested using an SVM classifier at different kernel functions at (P, R) = (20, 3). The linear kernel results in the best outcome on Messidor-2 as shown in Table 3.

**Table 3:** Results of Messidor-2 with SVM classifier and with different kernel functions, (P, R) = (20, 3)

SVM Kernel	Formula	Precision	Specificity	Recall (Sensitivity)	F1-score	Accuracy (Acc) (%)
Linear	$K\left({x}_{n},{x}_{i}\right)=\left({{x}_{n}}^{^{\prime}},{ x}_{i}\right)$	100%	100%	96.3%	0.9810	98.37
Gaussian RBF	$K\left({x}_{n},{x}_{i}\right)=\mathrm{exp}(-\gamma {\left|\left|{x}_{n}-{x}_{i}\right|\right|}^{2})$	100%	100%	94.4%	0.9710	97.70
*Polynomial*	$K\left({x}_{n},{x}_{i}\right)={\left.\left(1 + {{x}_{n,}}^{^{\prime}}{ x}_{i}\right.\right)}^{q}$	100%	100%	94.9%	0.9737	97.90
Gaussian	$K\left({x}_{n},{x}_{i}\right)=\mathrm{exp}(-\frac{||x\_n-x\_i ||^2}{2\sigma })$	100%	100%	93.8%	0.9679	97.61

To evaluate the effect of the value of R on the results, neighbourhood P is fixed at eight neighbours, and R equals one value of (2, 3, 4, 5). The results of applying ULBPEZ to Messidor-2 are presented in Table 4. The feature extraction method’s performance with R = 3 or 5 is much better than with R = 2 or 4. The focus will be on using R = 3 as it has the highest performance, and it is recommended to use the smaller R to decrease the block size as much as possible to detect features of small local details.

**Table 4:** Uniform ULBPEZ results on Messidor-2 using SVM with Linear kernel and P = 8

R	Precision (%)	Specificity (%)	Recall (Sensitivity) (%)	F1-score
2	84.9	92.3	61.3	0.7123
3	100	100	94.9	0.9740
4	98.6	99.3	68.2	0.8066
5	98.8	99.2	90.1	0.9422

**Fig. 7:**
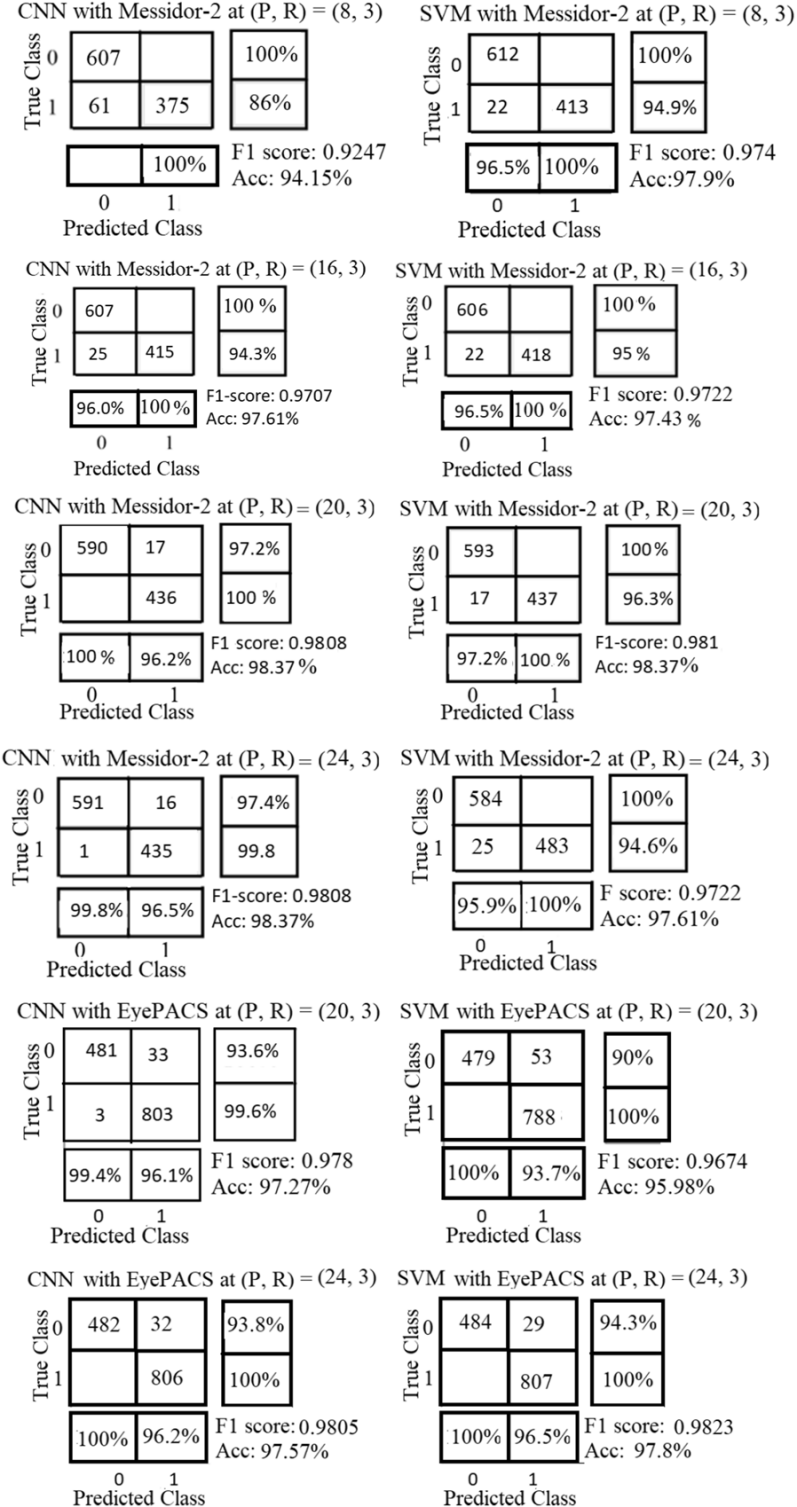
shows the confusion matrix results of ULBPEZ on Messidor2/EyePACS using the SVM and CNN model

The proposed uniform ULBPEZ has been evaluated on Messidor-2 and EyePACS using different circular neighbourhoods P and with the SVM "Linear" kernel and our CNN model. All results at neighbourhoods P = 8, 20, and 24 are acceptable performance on Messidor2 and all of them got the highest F1-score and AUC values with SVM or with the CNN model. The pairs (20, 3) and (24, 3) got the highest F1-score and AUC values on EyePACS. According to the EyePACS results, the pair (24,3) provides the best F1-scare. Figure 7 shows the confusion matrix of the binary classifier at four of the most successful pairings of (P, R). The performance of our proposed CNN is competing with SVM, as shown in Table 5.

**Table 5:**
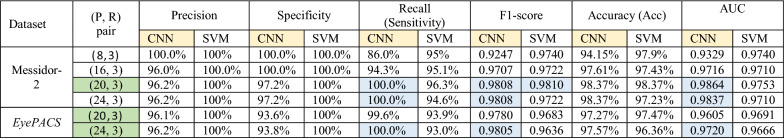
Classification into 2 classes results of ULBPEZ using SVM and CNN model

## Results of multi-classes classifier

**Fig. 8:**
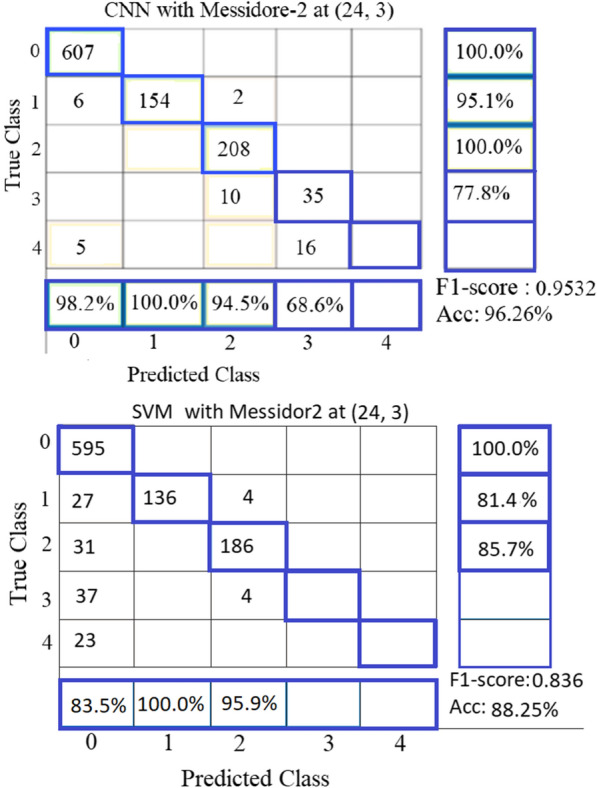
Confusion matrix results of grading DR on Messidor-2 at (P, R) = (24, 3): **a** Making use of the proposed CNN, **b** using SVM

**Fig. 9:**
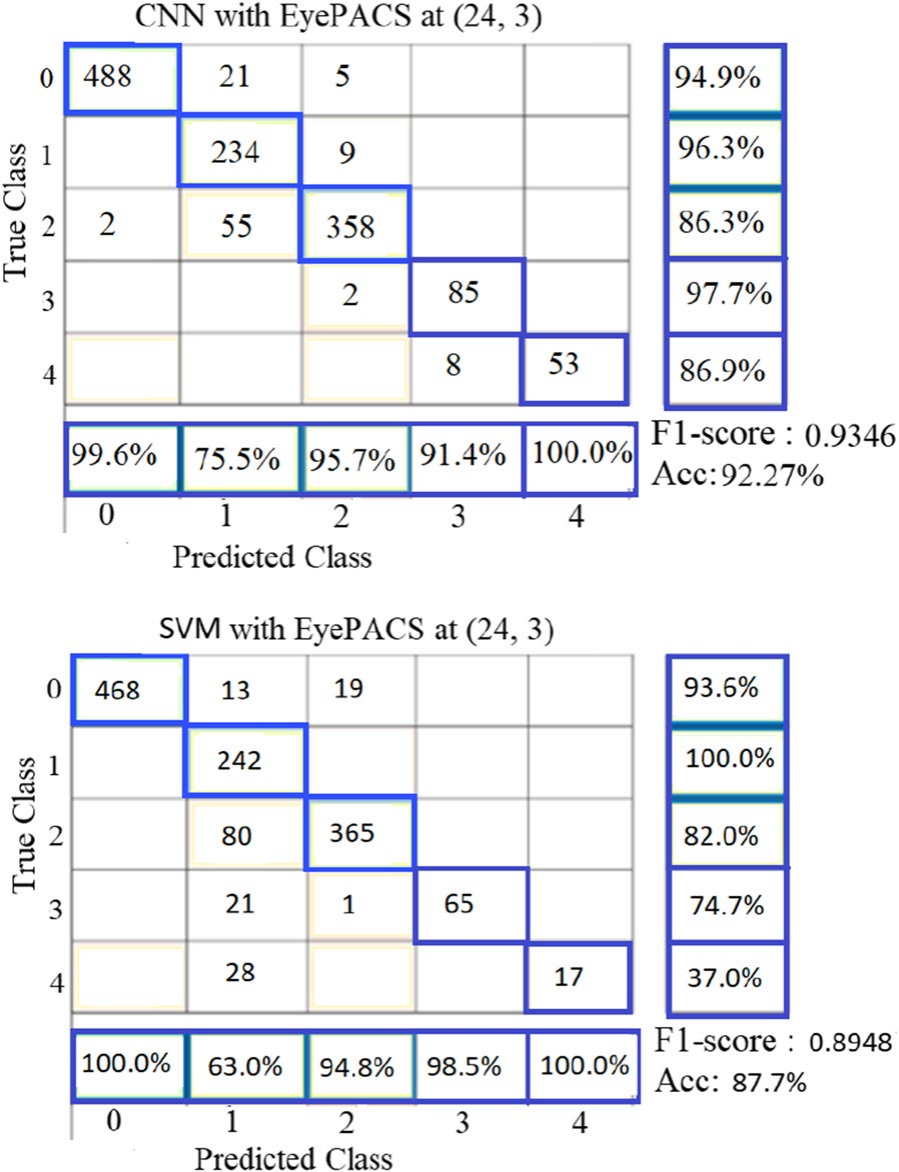
Confusion matrix results of grading DR on EyePACS at (P, R) = (24, 3) and with a doubling number of samples of grades (1:4): **a** Making use of the proposed CNN, **b** Using SVM

The best classification rate for grading DR into five classes on Messidor2 and EyePACS databases was at pair (P, R) = (24, 3) using the proposed CNN model and SVM. Figures 8 and 9 present the confusion matrixes of the obtained results at pair (24, 3). Figure 8 shows the failure of SVM to classify grade 3 (Severe Non-Proliferative DR (NPDR)) and grade 4 (Proliferative DR (PDR)) on Messidor2 and the failure of CNN to classify grade 4 on Messidor2. It is noticeable that in all databases of DR, the number of samples in grade 3 and grade 4 is too limited compared to grade 0, which has been classified correctly by 100%. The unbalanced results among different DR severity grades are because the number of samples of abnormal grades (1:4) is not enough for training the classifier properly. One of the solutions is to double the number of samples in each grade (1:4) by adding the vertical mirrored version of the images before processing them for feature extraction. Results showed that CNN model performance is much better than SVM in grading the severity of DR into 5 classes. After doubling the images of grades (1:4) in the EyePACS database, its size became 4400 images. That is the cause of getting balanced results among different DR severity grades with the EyePACS database as shown in Fig. 9.

Many researchers (Shaban et al. [26]; and David et al. [22]) regrouped the grades as a solution to the problem of the low classification rate of grades (2:4). We implemented the following two proposals for the new grouping: The first is our proposal. The grades of DR are divided according to three levels of severity: 0: “no DR,” 1: “mild,” and 5: “Moderate and Sever.” The images of old grades 2, 3, and 4 are grouped into one new grade named "5". This forms a database of the new three grades (0, 1, and 5). The second proposal for grading DR is proposed and presented by Shaban et al. [26]. The grades of DR are divided into three levels of severity (0, M, and S). Grade 0 means “No DR”. The samples from grades 1 “Mild”, and 2 “Moderate” are grouped into one grade named “Grade M”. The samples of grades 3 (Severe Non-Proliferative DR (NPDR)), and 4 (Proliferative DR (PDR)) are grouped into one grade named “Grade S”.

**Fig. 10:**
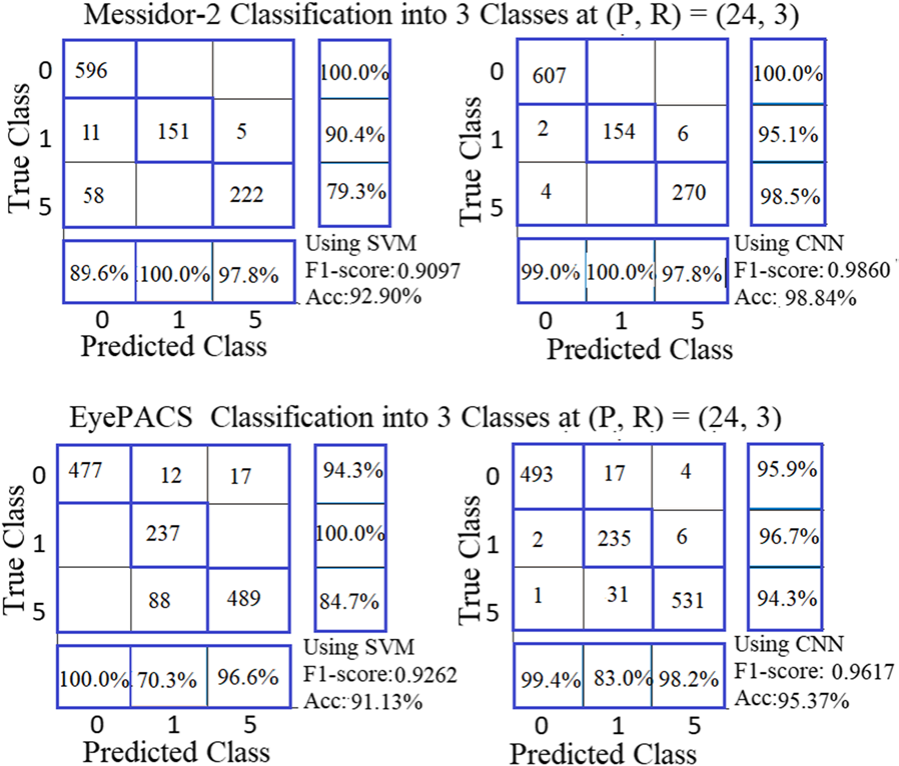
The best outcomes of ULBPEZ using SVM and our CNN model with grouping using our proposal

The DR diagnosis system performs well with our proposal of DR grading (0, 1, and 5) using SVM on Messidor-2 and EyePACS databases (see Fig. 10). From the results of the multiclass SVM classifier and grouping of the databases' grades according to our grouping proposal, the best pair is (24, 3) for EyePACS and Messidore-2 databases. The performance of the proposed CNN model outperforms SVM performance in classifying DR grades into 3 classes. The F1 score is 0.9860 with an accuracy of 98.84% for Messidore-2 and the F1 score is 0.9617 with an accuracy of 95.37% for EyePACS. Table 6 shows the details of classification into 3 classes of results using SVM and our proposed model.

**Table 6:**
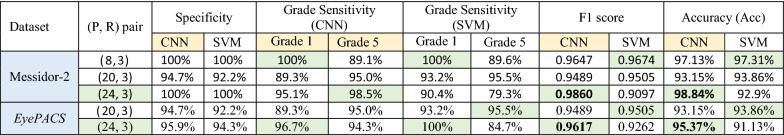
Classification into 3 classes results of ULBPEZ using SVM and CNN model

**Fig. 11:**
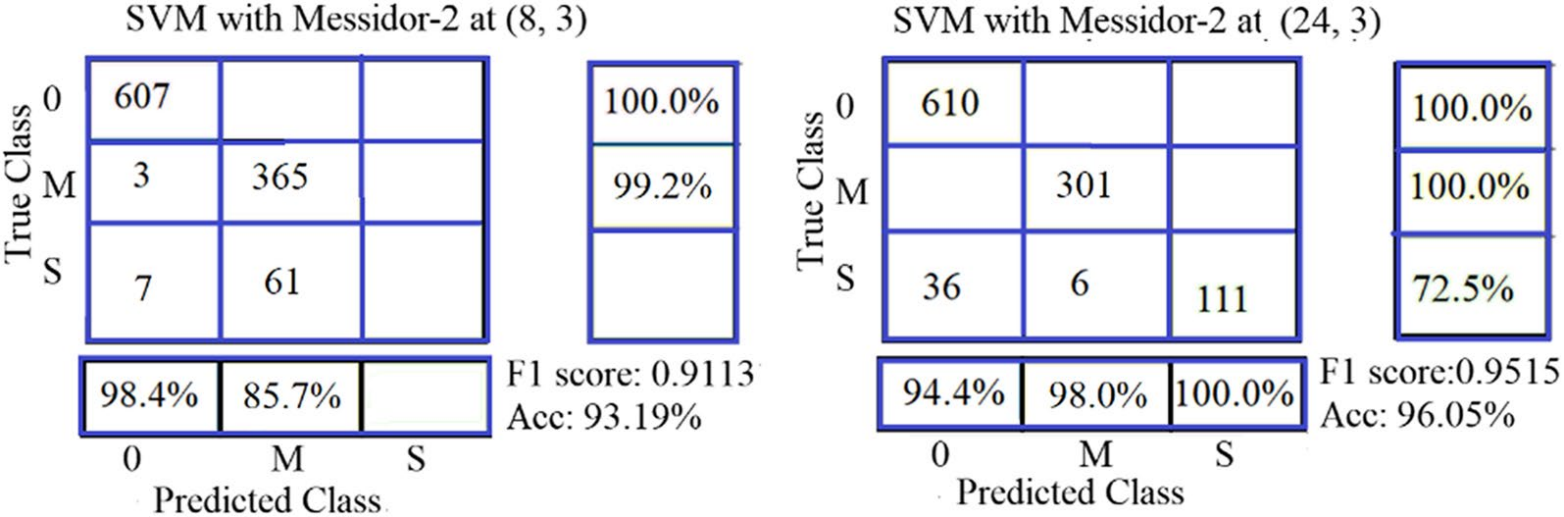
The best outcomes of SVM using ULBPEZ on Messidor-2 and EyePACS databases with grouping as Shaban et al. (2020) [26] proposal using SVM

Shaban et al. [26] proposal has limited success with the EyePACS database and failed with Messidore-2 to classify severe class (S) (see Fig. 11). It obtained an F1 score of 0.930 and an accuracy of 93.98% on the Kaggle EyePACS database.

## Comparison with other ultramodern techniques

The proposed method ULBPEZ applies the uniform LBP mapping features without performing any image segmentation step. It reduces the size of the features to a very small size. The achieved results outperform the results of comparable ultramodern techniques. To the best of the author’s knowledge, no other system analyses the texture of the retina background and detects DR without image segmentation. The proposed feature extraction method is novel and superior in producing very small feature sizes and it may be the best in its results scores compared with others that used the same database (Ramachandran et al. [17]; Johari et al. [20]; Lam et al. [21]; Usman et al. [30]; David et al. [22]; Gargeya and Leng [23]).

Some of these researchers (Ramachandran et al. [17]; Usman et al. [30]; David et al. [22]; Gargeya and Leng [23]) are using deep learning classifiers, feeding the deep learning classifiers with the image directly after some pre-processing. Table 7 summarises the performance, method, and database information of DR detection systems compared to the proposed method. This work showed that reliable computer diagnosis of DR could be accomplished for diabetics using robust enhancement steps and ULBPEZ.

**Table 7:** Performances of existing DR detection methods

Cited papers	Database	Methodology	Result
Ramachandran et al. [17]	Otago	Deep neural network software called Visiona	Sensitivity: 84.6%
	Messidor-1		Sensitivity: 96.0%
Johari et al. [20]	580 images Messidor-1	AlexNet	Acc = 88.3%
Manojkumar et al. [10]	NA	LBP is applied to each channel. The statistical features are calculated for each channel of the LBP image. A random forest algorithm is used for classification	True Positive Rate: 0.856 True Negative Rate: 0.897
Colomer et al. [3]	E-OPHTHA	LBPs and granulometric patterns	SVM: Acc range is (82.05%:85.33%) Gaussian processes: Acc: 87.62% Sensitivity: 83.48%
Wang et al. [15]	Messidor-1	R-FCN method by Dai et al. [16] after modification Classification of DR stages	Sensitivity: 92.59%
Li et al. [24]	Messidor-1	Grading DR severity using attention Deep Learning Network based on ResNet50	Acc: 92.6%, Sensitivity: 92.0%,
Shaban et al. [26]	EyePACS (3,648) images	The system outputs only three classes by merging mild and moderate in one class and severe NPDR and PDR in one class then using (CNNs) for classification to Grade DR severity	Sensitivity: 87%-89% Acc: 88%-89% for only 3-classes
David et al. [22]	Messidor-2	The system outputs only three classes by merging no DR, mild in one class and moderate and severe NPDR in one class, and PDR in one class then using (CNNs) for classification to Grade DR severity	Sensitivity: 96.8% for only 3-classes
Costa and Galdran [33]	Messidor-1	Grade DR severity using Multiple Instance Learning	AUC: 0.9
Dutta et al. [34]	EyePACS	Grading DR severity using VGGNet 16	Acc: 86.30%
Chetoui et al. [35]	Messidor-1	Detecting DR using CNNs (binary classifier) to normal and abnormal	AUC: 0.963
	EyePACS		AUC: 0.986, Sensitivity: 0.958
Kwasigroch et al. [36]	EyePACS	VGGNet Model	Acc: 81.70%, Sensitivity: 89.50%
Chowdhury et al. [37]	EyePACS	Inception v3 Model (binary classifier) in normal and abnormal	Acc: 61.3%
Sayres et al. [38]	EyePACS 2000 images	Grading DR severity using customized networks CNN	Acc: 88.4%, Sensitivity: 91.5%,
Sengupta et al. [39]	EyePACS	Inception-v3 Model	Acc: 90. 4%, Sensitivity: 90%
Pao et al. [40]	EyePACS	Bi channel customized CNN	Acc: 87.83%, Sensitivity:77.81% Specificity: 93.88%, AUC: 0.93
Samanta et al. [41]	EyePACS	DenseNet121 based	Acc: 84.1%
Thota and Reddy [42]	EyePACS	VGGNet Model	Acc: 74%, Sensitivity: 80.0% Specificity: 65.0%. AUC: 0.80
Ludwig et al. [43]	Messidor-2	Detect referral-warranted diabetic retinopathy (RDR) using DenseNet201	Acc: 87% Sensitivity: 80%
Proposed method ULBPEZ	Messidor-2	Methodology: Uniform LBP Encoded Zeros features (ULBPEZ)	
		Results of Binary Classifier:	
		(SVM) SE: 96.3%, F1-score: 0.9810, Acc: 98.37%, AUC: 0.9753 at (P, R) = (20, 3)	
		(SVM) SE: 94.6%, F1-score: 0.9722, Acc: 97.23%, AUC: 0.9710 at (P, R) = (24, 3)	
		(CNN) SE: 100.0%, F1-score: 0.9808, Acc: 98.37%, AUC: 0.9864 at (P, R) = (20, 3)	
		(CNN) SE: 100.0%, F1-score: 0.9808, Acc: 98.37%, AUC: 0.9837 at (P, R) = (24, 3)	
		Results of Multi-classes Classifier:	
		(CNN) Specificity: 100.0%, F1-score: 0.9860, Acc: 98.84% at (P, R) pair equal (24, 3)	
		(CNN) Specificity: 100.0%, F1-score: 0.9947, Acc: 97.13% at (P, R) pair equal (8, 3)	
	EyePACS	Methodology: Uniform LBP Encoded Zeros features (ULBPEZ)	
		Results of Binary Classifier:	
		(SVM) SE: 93.9%, F1-score: 0.9683, Acc: 97.47%, AUC: 0.9691 at (P, R) = (20, 3)	
		(CNN) SE: 100.0%, F1-score: 0.9805, Acc: 97.57%, AUC: 0.9720 at (P, R) = (24, 3)	
		Results of Multi-classes Classifier:	
		(CNN) Specificity: 95.9%, F1-score: 0.9617, Acc: 95.37% at (P, R) pair equal (24, 3)	

## Conclusion

In this paper, a new method for feature extraction from fundus images is presented and evaluated on the Messidor-2 and EyePACS databases for DR detection and grading. The proposed method differs from those in the literature in terms of the method of pre-processing and its novel technique to extract features. Compared to ultramodern feature extraction methods, the proposed method is superior to other methods as revealed by its small feature vector and its high score of accuracy and sensitivity. The used technique proved excellent for differentiating the normal and abnormal fundus image. The proposed system partially and incorrectly classifies severe NPDR, and PDR. Regrouping the DR grades into new grading (0: no DR, 1: mild, 5: moderate, severe NPDR, and PDR) enhances the outcome of grading and it differentiates excellence among the new grading 0, 1, 5. The differences in results between Messidore-2 and EyePACS databases due to differences in the quality of their images prove that we could get a high-performance DR diagnosing system if we could fix the quality of the fundus image by fixing all surrounding conditions (lighting, camera specification, focus, and exposure). Researchers should give more effort to the improvement of retinal fundus images for good DR screening. The proposed system outperforms the state of the artwork. The proposed CNN model performs better than SVM.

## Data Availability

The datasets of ULBPEZ images and extracted features generated during the current study are available in the following link https://elengmenofiaedu-my.sharepoint.com/:f:/g/personal/m_berbar_el-eng_menofia_edu_eg/EjpTo95CJj9Co8Qk_6M3W-kB6cqG9QvsQXR5VjwVnnds2A?e=Wr3vaH
